# Neuromuscular Adaptations Following Training and Protein Supplementation in a Group of Trained Weightlifters

**DOI:** 10.3390/sports6020037

**Published:** 2018-04-19

**Authors:** Christopher Taber, Kevin Carroll, Brad DeWeese, Kimitake Sato, Charles Stuart, Mary Howell, Kenton Hall, Caleb Bazyler, Michael Stone

**Affiliations:** 1 Department of Physical Therapy and Human Movement Science, Sacred Heart University, Fairfield, CT 06405, USA; 2 Center of Excellence for Sport Science and Coach Education, Department of Exercise and Sport Sciences, East Tennessee State University, Johnson City, TN 37614, USA; carrollkm1@gmail.com (K.C.); deweese@etsu.edu (B.D.); satok1@etsu.edu (K.S.); bazyler@etsu.edu (C.B.); STONEM@mail.etsu.edu (M.S.); 3 Department of Internal Medicine, Quillen College of Medicine, East Tennessee State University, Johnson City, TN 37614, USA; stuartc@etsu.edu (C.S.); howellm@etsu.edu (M.H.); hallh@etsu.edu (K.H.)

**Keywords:** protein, carbohydrate, weightlifting, supplementation

## Abstract

The purpose of this study was to examine the effects of a recovery supplement compared with a placebo on muscle morphology in trained weightlifters. Vastus lateralis and muscle fiber cross sectional area of type I and type II fibers were compared between groups using a series of 2 × 2 (group × time) repeated measure ANOVAs. Both groups on average improved cross-sectional area of the vastus lateralis, type I and type II muscle fibers from pre-to-post but individual response varied within both groups. Greater magnitude of changes in type I and type II muscle fibers were observed for the placebo group but not for vastus lateralis cross sectional area. Additionally, subjects were divided into large and small fiber groups based on combined fiber size at the start of the investigation. These findings indicate that the recovery supplement utilized provided no greater effect compared with a placebo in a 12-week block periodization protocol in trained weightlifters. The primary determinate of fiber size changes in the study was determined to be the initial fiber size of muscle fibers with larger practical changes observed in the small fiber group compared with the large fiber group in type I, II, and ultrasound cross-sectional area (CSA).

## 1. Introduction

Weightlifting is a weight class sport with competitors attempting to lift the most weight in two separate disciplines, the snatch and the clean and jerk [1]. In order to maximize performance, the muscular and nervous systems must synergistically coordinate to impart the high force and velocity necessary to lift the barbell successfully overhead. Initial improvements in strength training occur via neural mechanisms with muscular adaptations occurring at a slower rate [2,3]. As training progresses, adaptations in strength are more difficult to achieve and morphological adaptations within the muscle become critical [4]. Morphological changes, such as increased cross-sectional area (CSA), can be altered by resistance training [5,6,7]. These specific morphological changes within the muscle may also be impacted by nutritional countermeasures which can assist in tissue remodeling via increased synthesis of contractile proteins [8,9]. By combining resistance training and appropriate nutritional countermeasures, coaches and athletes may attempt to alter muscle architecture leading to increased performance.

Protein supplementation may provide additional enhancement in protein synthesis following exercise [10,11,12]. Evidence has been presented that protein intake can have direct effects on cellular signaling pathways that lead to accretion of the contractile proteins actin and myosin when combined with resistance exercise [13,14]. Protein intake simultaneous with resistance training has been shown to augment chronic adaptations mainly through increases in CSA and enhanced recovery from training [15]. By consuming adequate amounts of protein and carbohydrate following training athletes may improve recovery as well as facilitate the accretion of contractile proteins leading to increased muscle CSA. However, a paucity of research exists in trained athletes combining nutritional countermeasures with structured training plans.

Through the combination of resistance training and nutritional countermeasures (e.g., protein and carbohydrate supplementation), athletes strive to positively alter neuromuscular properties via increased CSA, specifically of type II muscle fibers. These nutritional countermeasures may provide a more robust protein synthetic response, facilitating the changes incurred following training and ultimately impacted performance. Therefore, the purpose of this paper is to investigate the effects of a carbohydrate and protein (CHO + prot) recovery beverage vs. a calorie free placebo on individual fiber changes, total muscle CSA and performance changes in trained weightlifters.

## 2. Materials and Methods

### 2.1. Materials

Vector red alkaline phosphatase substrate kits (SK-5105) and Vector SG peroxidase kits (SK-4705) were purchased from Vector Laboratories (Burlingame, CA, USA). Monoclonal anti-myosin (skeletal-fast) alkaline phosphatase (A4335) was purchased from Sigma (St. Louis, MO, USA). Monoclonal mouse anti-slow muscle myosin (MAB1628) was purchased from Millipore (Temecula, CA, USA).

### 2.2. Athletes

Ten trained male weightlifters participated in this study and were randomly assigned to a treatment or a placebo group. Subject descriptive data for the treatment and placebo group can be found in Table 1. Inclusion criteria required that each subject had been training regularly (four times a week minimum) for weightlifting competition for a minimum of one year and free of orthopedic injury for the past 6 months. Each subject read and signed a written informed consent form prior to beginning the study. This study was approved by the University’s Institutional Review Board. No athlete who had been training for less than a year on a periodized training protocol were admitted to this study. Only ten athletes at our research facility met the criteria for inclusion into this study. All other athletes were either untrained, did not meet the training history criteria, or had been injured in the past six months.

### 2.3. Experimental Design

A repeated measures design conducted in a double-blind fashion was used to determine if changes in muscle morphology were affected by a CHO + prot beverage taken immediately following training. Prior to training, subjects were randomly assigned to either the treatment group or a placebo group. The treatment group received a ready to drink CHO + prot beverage immediately following each workout and the placebo group consumed a calorie free beverage at the same time point. The CHO + prot fruit punch flavored recovery beverage contained 230 calories consisting of 16 g of hydrolyzed whey protein and 41 grams of carbohydrates consisting of sucrose and dextrose. (Gatorade, Chicago, IL, USA). The placebo group received an acaloric fruit punch drink (Great Value, Bentonville, AR, USA). The supplement and placebo beverages were placed in opaque shaker bottles with subject numbers placed on the lid of the bottle to ensure anonymity. Athletes were instructed to consume no additional supplements during the study and to refrain from eating for 30 min following the consumption of post workout beverage. Athletes were further instructed to maintain normal dietary practices and reminded consistently not to change their dietary habits over the course of the investigation.

### 2.4. Training Plan

A 12-week block periodized training program was completed for this study. Four blocks were completed consisting of a strength endurance block (3 weeks), strength block (4 weeks), power block (3 weeks) and a tapering and peaking block (2 weeks). Each athlete completed four training sessions per week consisting of general strength exercises, weightlifting movements and their derivatives. Training sessions followed a Monday, Wednesday, Thursday, Saturday schedule for the duration of the study. A detail of the training plan implemented can be found in Table 2, while the exercise-selection can be found in Table 3. Relative intensities (%1RM) were used to prescribe training loads, and were adjusted weekly using a sets and reps best system [16].

### 2.5. Muscle Biopsies

Percutaneous needle biopsies of vastus lateralis were performed using a 5-mm Bergstrom-Stille needle under suction after an overnight fast as previously described [17]. Muscle biopsies were obtained immediately pre and post intervention period. A 50- to 100-mg specimen was quickly blotted, and a portion was mounted on cork for sectioning. The remainder of the sample was frozen in an isopentane slurry cooled over liquid nitrogen. All the samples were then placed in liquid nitrogen and stored at −80 °C for later analysis.

### 2.6. Quantification of Muscle Fiber Type Composition and Fiber Size

Fiber composition was determined using methods described previously [18]. Muscle sections were stained for bright-field light microscopy in a two-step method using commercial monoclonal antibodies for fast and slow isoforms of myosin heavy chain. After acetone fixation and incubation with 1% normal rabbit serum, the slow myosin antibody was applied, followed by a peroxidase-conjugated rabbit anti-mouse IgG antibody. The fast myosin antibody was then applied. Slides were alcohol dehydrated, cleared with xylene, and preserved in synthetic medium. This technique allows discrimination of type I, type IIa, and type IIx a magnification of 4×. All sections were coded and then quantified independently by two observers who were unaware of which subject or treatment the image represented. Fiber diameter was measured using ImageJ version 1.49 (NIH, Bethesda, MD, USA) at 10× magnification for all slides. Twenty fibers of each fiber type were measured for diameter for each sample.

### 2.7. Muscle Architecture

A GE logiq P6 ultrasound (General Electric, Fairfield, CT, USA) was used to examine cross-sectional area (CSA) of the vastus lateralis for the duration of the study. The initial muscle biopsy incision site served as a landmark for ultrasound measurements and was used for pre and post measurement. A ML6-15, 7.5 MHz ultrasound probe was used for all measurements to capture images of the vastus lateralis on the corresponding leg used for the muscle biopsy.

Measurements were taken with the athletes rested on their side and hips perpendicular to the examination table in the axial plane with a knee angle of 120° ± 5° as measured by a goniometer [19]. During each measurement session the location was marked with a permanent marker and the probe was oriented perpendicular to the muscle for each sample. This mark also coincided with the initial muscle biopsy and was used as reference to ensure consistent marker placement.

CSA was measured by placing the probe on the muscle and moving it in the transverse plane to collect a cross-sectional image using the ultrasound device, as previously described by Bayzler et al. [20], The reliability of this method has been determined previously [21]. CSA was measured by tracing the inter-muscular interface in the cross-sectional images [21,22,23]. The examiner took three cross-sectional images from each sonogram. Images were collected pre-intervention and following the cessation of the training protocol coinciding with the week of the muscle biopsies. The means of CSA were assessed from the images and used for further analysis.

### 2.8. Performance Assessments

Countermovement jumps (CMJ) and isometric mid-thigh pulls were completed pre and post training to examine the effects of the training plan on athletic performance. Counter movement jumps were performed using a near weightless PVC pipe held on the upper back in the back squat position to standardize across participants and remove arm swing. Two trials were performed by each participant and one minute of rest was provided between repetitions.

Isometric mid-thigh pulls were completed after CMJ. The mid-thigh pulls were completed in a custom built isometric rack which allows for consistent and standardized knee and hip angles. Prior to the first testing session all subjects were measured to ensure a knee angle of 125° and hip angle of 145° using a goniometer. The subjects then performed two warm-up trials with 50% and 75% of their perceived maximal effect with one minute of rest between each trial. Following the second rest period subjects performed two maximal effort trials.

The CMJ and mid-thigh pulls were performed on dual force plates (2 separate 45.5 × 91 cm force plates; RoughDeck HP, Rice Lake, WI, USA) sampling at 1000 Hz. Variables calculated for vertical jumps were peak power allometrically scaled to body mass (W·BM^^2/3^). Variables calculated for isometric mid-thigh pulls was isometric peak force allometrically scaled to body mass (N·BM^^2/3^).

### 2.9. Statistical Analysis

Intraclass correlation coefficients (ICC) were used to determine the test-test reliability between images of the ultrasound CSA. Following Shapiro-Wilks normality and Levene’s equality of variance calculations, a 2 × 2 (group × time) repeated measures analysis of variance (ANOVA) was used to compare treatment and placebo groups for changes in CSA. A 2 × 2 (group × time) mixed design ANOVA was used to compare muscle fiber size changes from immunohistochemistry samples. Additionally, Pearson product-moment correlations were calculated between baseline morphology measurements and the change scores through intervention, to assess the impact these variables had on the outcomes of the study. Following this analysis, subjects were regrouped evenly based on starting fiber size (i.e., a small and a large group) and additional 2 × 2 (group × time) mixed design ANOVAs were performed. Specifically, we used a pooled fiber size for each subject (combined Type I and Type II size) to perform this grouping. Any significant main effect in the ANOVA was followed with a Holm-Bonferroni post-hoc adjustment to further examine the statistical significance. Effect sizes were generated and interpreted as trivial, small, moderate, large, very large, and nearly perfect when Cohen’s d was 0.0, 0.2, 0.6, 1.2, 2.0, and 4.0 based on the scale by Hopkins [24]. All statistical analyses were performed with JASP version 0.8.1.2 (JASP team 2017, jasp-stats.org) and statistical significance for all analyses was set at *p* ≤ 0.05.

## 3. Results

**Figure 1:**
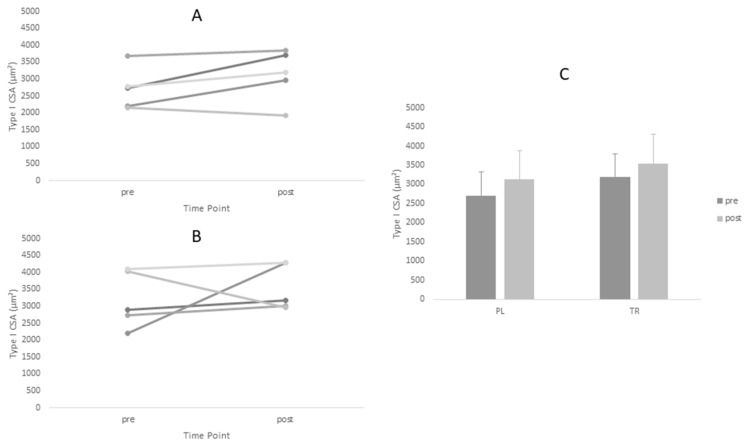
Changes in Type I CSA for Treatment and Placebo Groups Pre and Post Intervention Period. (**A**) Treatment group; (**B**) Placebo group; and (**C**) Group means.

**Figure 2:**
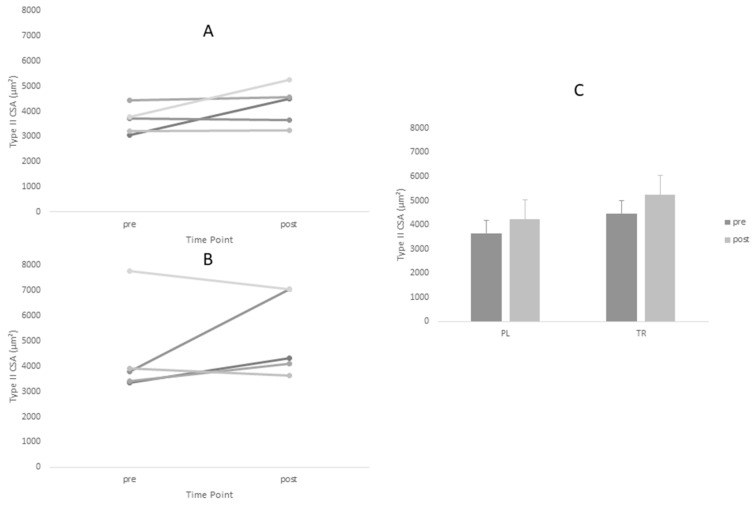
Changes in Type II cross-sectional area (CSA) for Treatment and Placebo Groups Pre and Post Intervention Period. (**A**) Treatment group; (**B**) placebo group; and (**C**) group means.

**Figure 3:**
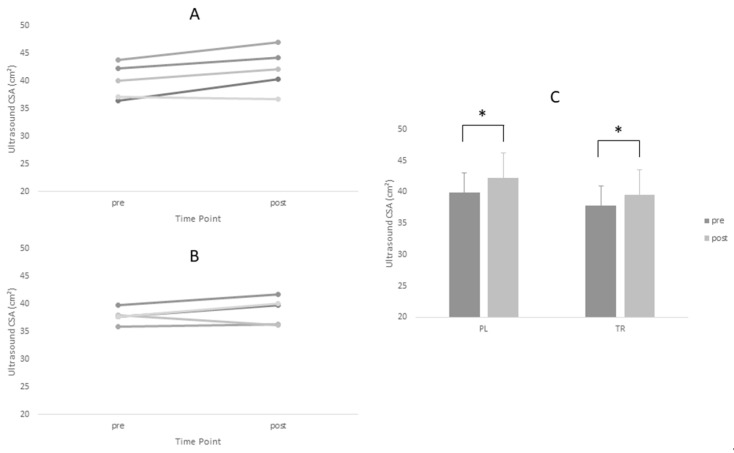
* = main time effect. Changes in Vastus Lateralis CSA for Treatment and Placebo Groups Pre and Post Intervention Period. (**A**) Treatment group; (**B**) placebo group; and (**C**) group means.

Descriptive statistics for all analyzed variables are displayed in Table 4. Adequate test-retest reliability of ultrasound CSA measurements were observed (ICC = 0.96). Tests of normality revealed a statistical deviation from the normal distribution in the TR group for post-intervention values of Type I CSA and pre-intervention values of Type II CSA. ANOVA yielded no significant main effects for Type I or Type II fiber size. However, a significant time main effect was observed for ultrasound CSA (*p* = 0.005). Post-hoc tests revealed no between-group differences at either time point for ultrasound CSA (*p* > 0.05). Based on effect size, the PL group increased fiber size measurements (Type I *d* = 0.60, Type II *d* = 0.88) to greater magnitude than TR group (Type I *d* = 0.45, Type II *d* = 0.45) (Figure 1 and Figure 2, Table 4).The opposite was true of ultrasound CSA measurements where the TR group produced greater effect magnitudes (*d* = 1.08) than the PL group (*d* = 0.64) (Figure 3, Table 4). There were no statistically significant main effects for either IPFa or CMJ PPa (*p* > 0.05).

Moderate negative correlations existed between Type I baseline and Type I size change (*r* = −0.579, *p* = 0.079), between Type II baseline and Type II size change (*r* = −0.441, *p* = 0.202), but not between ultrasound CSA baseline and ultrasound CSA change (*r* = 0.352, *p* = 0.318). To further examine the effects of baseline muscle size, ANOVA revealed no main effects for Type I fiber size (group main effect, *p* = 0.059), Type II fiber size (time main effect, *p* = 0.091). However, a time main effect was observed for ultrasound CSA (*p* = 0.018). Post-hoc testing did not reveal any additional significant between-group effects (*p* > 0.05). Effect size supported the hypothesis that smaller baseline size resulted in greater size changes. The small group increased size of Type I (*d* = 1.16), Type II (*d* = 0.99), and ultrasound CSA (*d* = 0.75). The large group resulted in effect sizes of Type I (*d* = −0.02), Type II (*d* = 0.20), and ultrasound CSA (*d* = 0.29). No statistically significant main effects were observed for IPFa or CMJ PPa in this analysis (*p* > 0.05), however there was a large effect size increase for CMJ PPa in the large group (*d* = 1.24), while only a trivial change for the small group (*d* = 0.06).

## 4. Discussion

This study compared the effects of a CHO + prot recovery beverage versus a calorie free placebo on muscle morphological changes in trained weightlifters. Although we observed no statistically significant alterations in muscle morphology or performance resulting from CHO + prot or placebo supplementation, it was intriguing that baseline muscle fiber size seemed to have an impact on the result. Both groups improved from pre-to-post on measurements of type I, type II, and ultrasound CSA. The PL group increased fiber CSA to a greater extent than did the TR group, based on effect magnitudes. Conversely, the ultrasound CSA enhancements supported the TR group, although each group had a moderate effect size (TR *d* = 1.08, PL *d* = 0.64). These slight divergences in effect magnitudes are likely driven in part by the relatively large standard deviations observed (Table 4), or by the lack of a control group. Additionally, several variables in the current study were not normally distributed. This is likely a common occurrence on sport teams, where one or more athletes might be physiologically more advanced than the team average. However, it is important to consider this when interpreting the results of the study, particularly ANOVA results. For example, the fiber CSA changes favored the PL group based on effect magnitudes. However, when examining the percent-changes for each respective group, there is little difference. The standard deviations were larger for TR group fiber CSA compared to the PL group. These heterogeneous responses should be considered when interpreting the results of this investigation. Allometrically scaled IPF and CMJ PP increased for the whole cohort on average (IPFa = 3.4 ± 10.4% increase, CMJ PPa = 8.2 + 11.8% increase), while there was a slight favoring of the PL group for IPFa based on effect size (*d* = 0.41). Both groups enhanced CMJ PPa similarly (TR *d* = 0.64, PL *d* = 0.61) suggesting that the CHO + prot supplementation did not impact the outcomes of these variables.

The moderate negative correlations (*r* = −0.441 and −0.579) between baseline fiber size and the change in fiber size seem to suggest that these baseline values inherently effected the outcome of the study. This result is not surprising, as it is likely the smaller subjects had a higher propensity to grow during the short duration of the study. Subjects who were already significantly hypertrophied prior to the intervention, alternatively, had less hypertrophic potential. It is possible that the well-trained status of the subjects in our investigation affected the results concerning CHO + prot supplementation. Further investigation revealed when the subjects were regrouped into either small or large baseline muscle size groups that the small group increased size more so than the large group. The small group’s increase in Type I, Type II, and ultrasound CSA with moderate effect magnitudes were greater than the trivial-to-small effects noted for the large group. Interestingly, the large group increased CMJ PPa with a large effect magnitude (*d* = 1.24) compared to a trivial effect for the small group (*d* = 0.06). This is surprising as when the groups were separated based on baseline muscle size the results seemed to favor the small group. Perhaps this result can be explained by other adaptive mechanisms separate from muscle morphological changes (e.g., neural adaptations, tendon adaptations, metabolic efficiency, etc.) obtained by the large group through training. Although beyond the scope of the current study, the large group’s substantially hypertrophied muscles pre-study may have allowed their adaptations to skew towards more functional enhancements rather than morphology alone.

Compared to pre-study measurements, both groups improved muscle fiber size of type I and type II fibers with no main effect present for between group comparisons. Studies examining supplementation around the workout window have displayed contradictory outcomes in type I and type II muscle fiber measurements. Andersen et al. [25], found increases in type I and II fibers in only the protein group whereas Hartman et al. [26], and Cribb & Hayes found increases in type II fibers only with protein supplementation groups [27]. These studies used untrained and moderately trained individuals which may display differing responses to resistance training and supplementation compared to trained athletes. In previous investigations, which investigated effects of supplementation in trained males, Hoffman et al. [28], failed to demonstrate a significant difference between treatment and control groups. The current study found similar results where CHO + prot supplementation in trained weightlifters did not produce statistically significant results compared with a placebo group.

Though this study was, to an extent, limited by subject size, it contained a homogenous group of highly-trained individuals who had a similar training background. Practically, the inclusion of a CHO + prot recovery beverage conferred no greater benefits at the fiber or whole muscle level compared with a placebo. However, this does not indicate that CHO + prot supplementation is not beneficial, but rather muscle size in our group of highly-trained weightlifters did not change according to supplementation. This study was limited to 12 weeks in length where adaptations caused by training and supplementation may not come to fruition until cellular proteins have developed in sufficient amounts resulting larger scale, structural changes. Future investigations should attempt to look at long term supplementation over the course of an annual plan and combine supplementation with trained athletes in order to access effectiveness for sporting performance.

## 5. Conclusions

The protein and carbohydrate recovery supplement did not improve CSA or type I and type II fiber size compared to a placebo in trained weightlifters, although both groups did increase size. The changes observed in the study were more related to initial fiber size with the smaller fiber size subjects displaying greater changes than those with larger baseline fiber sizes. Both groups improved from pre-training to post-training with greater fiber and total CSA, without between group statistical differences. From a practical standpoint, inclusion of CHO + prot supplement may not have a large effect on muscle fiber size in the short term compared with a placebo in highly-trained weightlifters.

## Figures and Tables

**Table 1:** Subject Descriptive Data.

	Treatment	Placebo
*n*	5	5
Age (years)	28.4 ± 5.4	33.7 ± 3.2
Height (cm)	179.6 ± 4.5	175.2 ± 2.8
BM (kg)	95.3 ± 12.3	93.5 ± 15.3
Training Age (years)	5.2 ± 3.2	4.9 ± 3.4
EST 1RM	170.6 ± 31.8	155 ± 38.9
EST 1RM STR/BW	1.8 ± 0.3	1.7 ± 0.3

Note: Values are means ± standard deviations, EST 1RM = Back squat 1 repetition maximum, EST 1RM STR/BW = ratio of back squat to body mass.

**Table 2:** 12 Week Training Plan.

Week	Sets × Reps	Dailey Intensities
1	3 × 10	M, M, L, L
2	3 × 10	MH, MH, ML, ML
3	3 × 10	H, H, L, VL
4	3 × 5 (1 × 5)	ML, ML, L, VL
5	5 × 5	M, M, ML, ML
6	3 × 3 (1 × 5)	MH, MH, VL, L
7	3 × 2 (1 × 5)	ML, M, ML, L
8	5 × 5	H, MH, ML, L
9	3 × 3(1 × 5)	MH, M, L, L
10	3 × 2(1 × 5)	ML, L, VL, Meet
11	3 × 5	M, M, ML
12	3 × 5	L, L, VL

Note: Repetition maximums based on sets and reps best system. (DeWeese, Same & Serrano, 2014) VL = very light, L = light, ML = medium light, M = medium, MH = medium heavy, H = heavy, Meet = competition day.

**Table 3:** Exercise Selection.

Weeks	Exercises: Mon & Thurs	Wednesday	Saturday
1–3	BSQ	SN	SGS
	SP	CGS	SN
	DBP	CPP	SDL
		CDL	DBR
		DBR	
4–7	BSQ	SN	SGSS
	PP	CGS	SN
	BNP	CGBK	CJ
	DBP	CDL	SDL
		CGR	SGR
8–10	BSQ	SN	SGS
	JRK	CGS	SN
	DBP	SGP	CJ
		CDL	SDL
11–12	BSQ	PS	
	DBP	CGS	COM
	FRR	CGS	
		SLDL	
		DBR	

Note: COM = Competition, BSQ = back squat, JRK = Jerk, SP = strict press, PP = push press, BNP = behind neck press, DBP = dumbbell press, FRR = front raise, CGS = clean grip shoulder shrug, SGS = snatch grip shoulder shrug, DBR = dumbbell row, CPP = clean grip pull from power positon, CBK = clean grip pull below the knee, SGP = snatch grip pull from floor, CDL = clean grip stiff leg deadlift, SDL snatch grip stiff leg deadlift, GCR = clean grip row, SGR = snatch grip row, PS = power snatch, SN = Snatch, CJ = clean and jerk.

**Table 4:** Descriptive Data for Muscle Size Measurements and Performance Changes.

Conditon	Group	Pre	Post	%Change
US CSA (cm^2^)	Treatment	37.8 ± 1.4	38.8 ± 2.5	2.6 ± 4.7
	Placebo	39.9 ± 3.2	42.0 ± 3.9	5.4 ± 4.3
	Total	38.8 ± 2.5	40.4 ± 3.5 *	4.0 ± 4.5
Type I CSA (μm²)	Treatment	3194.9 ± 838.3	3541.3 ± 680.9	18.3 ± 44.8
	Placebo	2710 ± 611.2	3123.4 ± 761.0	15.7 ± 19.9
	Total	2952.5 ± 737.4	3332.3 ± 715.5	17.0 ± 32.7
Type II CSA (μm²)	Treatment	4446.8 ± 1869.9	5238.8 ± 1681.8	23.9 ± 38.8
	Placebo	3649.2 ± 540.54	4244.0 ± 792.62	17.4 ± 23.3
	Total	4048.0 ± 1364.0	4741.4 ± 1345.8	20.7 ± 30.4
IPFa (N∙bm^^2/3^)	Treatment	249.2 + 42.1	248.8 + 36.8	0.2 + 5.2
	Placebo	214.7 + 32.3	227.4 + 30.2	6.7 + 13.7
	Total	231.9 + 39.8	238.1 + 33.7	3.4 + 10.4
CMJ PPa (W∙bm^^2/3^)	Treatment	237.2 + 24.8	256.6 + 34.6	9.0 + 17.4
	Placebo	219.3 + 25.8	235.0 + 25.5	7.3 + 3.1
	Total	228.3 + 25.6	245.8 + 30.8	8.2 + 11.8

Note: All data are means ± standard deviation, US = ultrasound, CSA = cross-sectional area, IPFa = Allometrically scaled Isometric Peak force, CMJPPa = Countermovement jump Peak Power allometrically scaled, * = main time effect.
